# Medical Image Registration Algorithm Based on Bounded Generalized Gaussian Mixture Model

**DOI:** 10.3389/fnins.2022.911957

**Published:** 2022-06-02

**Authors:** Jingkun Wang, Kun Xiang, Kuo Chen, Rui Liu, Ruifeng Ni, Hao Zhu, Yan Xiong

**Affiliations:** ^1^Department of Orthopaedics, Daping Hospital, Army Medical University, Chongqing, China; ^2^College of Automation, Chongqing University of Posts and Telecommunications, Chongqing, China; ^3^School of Software Engineering, Chongqing University of Posts and Telecommunications, Chongqing, China

**Keywords:** medical image registration, gray-level-based registration, multimodal, Gaussian mixture model, bounded generalized Gaussian mixture model

## Abstract

In this paper, a method for medical image registration based on the bounded generalized Gaussian mixture model is proposed. The bounded generalized Gaussian mixture model is used to approach the joint intensity of source medical images. The mixture model is formulated based on a maximum likelihood framework, and is solved by an expectation-maximization algorithm. The registration performance of the proposed approach on different medical images is verified through extensive computer simulations. Empirical findings confirm that the proposed approach is significantly better than other conventional ones.

## Introduction

Image registration is an essential part of computer vision and image processing (Visser et al., 2020), which is widely used in medical image analysis and intelligent vehicles (Zhu et al., 2013, 2017, 2021a,2021b,^2022^). Medical image analysis is the basis for judging the patient’s condition in future intelligent diagnosis and treatment or auxiliary diagnosis and treatment (Weissler et al., 2015; Yang et al., 2018). More importantly, image registration sets the stage for subsequent image segmentation and fusion (Saygili et al., 2015; Zhu et al., 2019). Current clinical practice typically involves printing images onto radiographic film and viewing them on a lightbox. The computerized approach offers potential benefits, particularly by accurately aligning the information in different images and providing tools to visualize the composite image. A key stage in this process is the alignment or registration of the images (Hill et al., 2001).

The premise of image registration is that there is a same logical part between the reference image and the floating image (Gholipour et al., 2007; Reaungamornrat et al., 2016). Image registration realizes transformation by determining the space coordinate transformation between two image pixels, which enables the corresponding region on the reference image to coincide with the floating image in space (Zhang et al., 2019). This means that the same anatomical point on the human body has the same spatial position (the same position, angle and size) on two matched images (Gefen et al., 2007).

There are two medical image registration methods: feature-based registration and gray-level-based registration (Sengupta et al., 2021). The feature-based registration method does not directly utilize the gray-level information of the image. It is based on abstracting the geometric features (such as corners, the center of the closed region, edges, contours, etc.) that remain unchanged in the image to be registered. The parameter values of the transformation model between the images to be registered are obtained by describing the features of the two images, respectively, and establishing the matching relationship (Huang, 2015). The image registration based on this feature has advantages of less computation and faster registration speed, and it is robust to changes of gray image scale. However, its registration accuracy is usually not as high as that of gray-level-based image registration (Li et al., 2020; Ran and Xu, 2020).

In the gray-level-based medical image registration method, a similarity measure function between images is established through the gray information of the entire image (Yan et al., 2020). The transformation model parameters between images are obtained by maximizing and minimizing the value of the similarity measure function (Zhang et al., 2019). The gray-level-based image registration algorithm uses all the gray information of the image in the registration process. Therefore, the precision and robustness of the obtained transformation model are higher than the feature-based image registration (Frakes et al., 2008). The commonly used gray-level-based image registration methods are sequential similarity detection algorithm (SSDA), cross-correlation, mutual information, and phase correlation (Gupta et al., 2021). Based on the traditional algorithms, Yan et al. (2010) extracted a fast and effective algorithm, SSDA. Anuta (1970) proposed an image registration technique using Fourier transform for cross-correlation image detection and calculation to improve speed performance of registration. Evangelidis and Psarakis (2008) offered a modified version of the correlation coefficient as a performance criterion for image approval. Zheng et al. (2011) proposed a cross-correlation registration algorithm based on image rotation projection to avoid rotation and interpolation steps in image registration, reducing data dimension and computational complexity. For image registration using mutual information as a similarity measure, Pluim et al. (2000) combined image gray level with spatial image information and added image gradient into the algorithm, which successfully solved the problem of finding the global optimal solution in the registration process. A direct image registration method using mutual information (MI) as an alignment metric was proposed by Dame and Marchand (2012). A set of two-dimensional motion parameters can be estimated accurately in real time by optimizing the maximum mutual information. Lu et al. (2008) proposed a new joint histogram estimation method, which utilizes Hanning’s windowed since approximation function as a kernel function of partial volume interpolation. Orchard and Mann (2009) utilized the maximum likelihood clustering method of the joint strength scatter chart. The expected probability of the cluster is modeled as a Gaussian mixture model (GMM), and the expectation-maximization (EM) method is utilized for achieving solution in iterative algorithm. Sotiras et al. (2013) emphasized the technology applied to medical images and systematically presented the latest technology. The paper provided an extensive account of registration techniques in a systematic manner. Pluim et al. (2004) compared the performance of mutual information as a registration measure with that of other *f*-information measures. An important finding is that several measures can potentially yield significantly more accurate results than mutual information. Klein et al. (2007) compared the performance of eight non-rigid registration optimization methods of medical images. The results show that the Robbins–Monro method is the best choice in most applications. With this approach, the computation time per iteration can be lowered approximately 500 times without affecting the rate of convergence. However, the distribution range of GMM is (−∞, + ∞), and so the method could not process the target information in a fixed area.

In the field of computer vision, image pixel values are distributed over a limited area of [0, 255]. Therefore, the bounded generalized Gaussian mixture model (BGGMM) is used to model the image (Nguyen et al., 2014), which can more thoroughly describe the joint intensity vector distribution of the image pixels and highlight the details of the image. The BGGMM has good robustness at the same time. Therefore, based on the BGGMM, this paper models both single-modality and multimodal image registration and then solves the model under the framework of maximum likelihood estimation (Zhu and Cochoff, 2002). Experimental verification results on a large number of image data sets show that compared with the existing gray-level-based medical image registration algorithm based, the image registration accuracy of the proposed method is improved.

## Problem Formulation

Suppose that two different medical images are registered, one medical image represents the reference image, denoted by A, and the other represents the floating image, denoted by B. These two different medical images come from different sensors. Therefore, each pixel position x in the space of two medical images corresponds to a pixel value, and we use the joint intensity vector to represent the intensity value of the two images at the position. Here, *I_x_* can be expressed as:


(1) $$I_{x}=\left[A_{x};B_{x}\right]$$

Among them, *A_x_* and *B_x_*, respectively, represent the pixel value of the reference image and the floating image at the pixel position *x*. In order to realize the registration of two images, it is necessary to assign *N* registration parameters to each image to describe the spatial transformation of the image. θ can represent the set of all registration parameters. Then, the joint intensity vector of the registration image after employing registration parameters can be re-expressed as $I_{x}^{\theta }$.

The bounded generalized Gaussian mixture model (BGGMM) is used to describe the distribution of the joint intensity. The probability distribution of the joint strength vector is:


(2) $$p\,\left(I_{x}^{\theta }|\rho \right)=\sum_{m=1}^{M}\tau_{m}\,B\,G\,\left(I_{x}^{\theta }|u_{m},\sigma_{m},\Lambda_{m}\right)$$

Where ρ={*u*_*m*_,σ_*m*_,Λ_*m*_,τ_*m*_}is the model parameters, *M* represents the number of bounded generalized Gaussian (BGG) distribution components in the mixture model, *u_m_*, σ_*m*_ and Λ_*m*_, respectively, represent the mean, covariance, and shape parameters of the *m*-th BGG distribution component. τ_*m*_represents the weight of the distribution component in the mixture model and satisfies the condition τ_*m*_≥0 and $\sum_{m=1}^{M}\tau_{m}=1$. *BG*(.) represents a BGG distribution, i.e.,


(3) $$B\,G\,\left(I_{x}^{\theta }|u_{m},\sigma_{m},\Lambda_{m}\right)=\frac{T\,\left(I_{x}^{\theta }|u_{m},\sigma_{m},\Lambda_{m}\right)\,H\,\left(I_{x}^{\theta }|\Omega_{m}\right)}{\int_{\partial_{m}}\text{T}\,\left(I_{x}^{\theta }|u_{m},\sigma_{m},\Lambda_{m}\right)\,d\,x}$$

Which ∂*_m_* represents a bounded support area, and the distribution $\text{T}\,\left(I_{x}^{\theta }|u_{m},\sigma_{m},\Lambda_{m}\right)$ is written as


(4) $$T\,\left(I_{x}^{\theta }|u_{m},\sigma_{m},\Lambda_{m}\right)=\alpha \,\left(\Lambda_{m}\right)\,\exp\left(-\beta \,\left(\Lambda_{m}\right)\,{\left|\frac{I_{x}^{\theta }-u_{m}}{\sigma_{m}}\right|}^{\Lambda_{m}}\right)$$

and


(5) $$H\,\left(I_{x}^{\theta }|\Omega_{m}\right)=\left\{ \begin{array}{c} 1,i\,f\,I_{x}^{\theta }\,b\,e\,l\,o\,n\,g\,s\,t\,o\,\partial_{m}\\ 0,o\,t\,h\,e\,r\,w\,i\,s\,e \end{array}\right.$$

(6) $$\alpha \,\left(\Lambda_{m}\right)=\frac{\Lambda_{m}\,\sqrt{\Gamma \,\left(3/\Lambda_{m}\right)}}{2\,\sigma_{m}\,\Gamma \,\left(1/\Lambda_{m}\right)\,\sqrt{\Gamma \,\left(1/\Lambda_{m}\right)}},\beta \,\left(\Lambda_{m}\right)={\left[\frac{\Gamma \,\left(3/\Lambda_{m}\right)}{\Gamma \,\left(1/\Lambda_{m}\right)}\right]}^{\Lambda_{m}/2}$$

Where Γ(⋅) is the gamma function.

Therefore, *X* represents the number of pixels, and the log-likelihood function of image registration is:


(7) $$ℒ\,\left(\rho \right)=\sum_{x}^{X}l\,o\,g\,p\,\left(I_{x}^{\theta }|\rho \right)$$

In the framework of maximum likelihood, the hidden variable *z*_*xm*_ that is introduced to the model indicates the category of the cluster that $I_{x}^{\theta }$ belongs to, that is, it belongs to the *m*-th (BGG) distribution component. Therefore, the log-likelihood function of the model can be written as:


(8) $$ℒ\,\left(\rho \right)=\sum_{x}^{X}l\,o\,g\,p\,\left(I_{x}^{\theta },z_{x\,m}|\rho \right)$$

## Parameters Estimation

### Density Estimation

According to the above model, the EM algorithm is used to estimate various parameters involved in the model. The EM algorithm is mainly divided into two steps, step E and step M.

Step E: $Q\,\left(\rho ,\rho^{t}\right)=E\,\left[ℒ\,\left(\rho \right)|I_{x}^{\theta },\rho^{t}\right]$

Step M: $\rho^{t+1}=\max_{\rho }Q\,\left(\rho |\rho^{t}\right)$

Here *t* represents the *t*-th iteration. The final model parameters can be determined by iterating these two steps.

In step E, the probability that $I_{x}^{\theta }$ belonging to the *m*-th cluster is given:


(9) $$\eta \,\left(z_{x\,m}\right)=p\,\left(z_{x\,m}|I_{x}^{\theta },\rho \right)=\frac{\tau_{m}\,B\,G\,\left(I_{x}^{\theta }|u_{m},\sigma_{m},\Lambda_{m}\right)}{\sum_{m=1}^{M}\tau_{m}\,B\,G\,\left(I_{x}^{\theta }|u_{m},\sigma_{m},\Lambda_{m}\right)}$$

Where $\sum_{m=1}^{M}\eta \,\left(z_{x\,m}\right)=1$. Using the posterior distribution η(*z*_*xm*_) and the current parameters ρ^(*t*)^


$$Q\,\left(\rho ,\rho^{t}\right)=E\,\left[ℒ\,\left(\rho \right)|I_{x}^{\theta },\rho^{t}\right]$$

$$\;\;=\sum_{x=1}^{X}\sum_{m=1}^{M}\eta \left(z_{x\,m}\right)$$

(10) $$\left[\begin{array}{c} \log\tau_{m}+\log T\,\left(I_{x}^{\theta }|u_{m},\sigma_{m},\Lambda_{m}\right)+\\ \log H\,\left(I_{x}^{\theta }|\Omega_{m}\right)-\log\,\int_{\partial }T\,\left(I_{x}^{\theta }|u_{m},\sigma_{m},\Lambda_{m}\right)\,d\,x \end{array}\right]$$

At step M, the parameters $u_{m}^{t+1},\sigma_{m}^{t+1},\Lambda_{m}^{t+1},\tau_{m}^{t+1}$ at the time (*t*+1) are updated by the maximizing equation (10). The results are as follows:


(11) $$u_{m}^{t+1}=\frac{\sum_{x=1}^{X}\eta \,\left(z_{x\,m}\right)\,\left({\left|I_{x}^{\theta }-u_{m}^{t}\right|}^{\Lambda_{m}^{t}-2}\,I_{x}^{\theta }+R_{m}\right)}{\sum_{x=1}^{X}\eta \,\left(z_{x\,m}\right)\,{\left|I_{x}^{\theta }-u_{m}^{t}\right|}^{\Lambda_{m}^{t}-2}}$$

Where *R_m_* represents:


(12) $$R_{m}=\frac{\sum_{o=1}^{O}s\,i\,g\,n\,\left(u_{m}^{t}-S_{o\,m}\right)\,{\left|S_{o\,m}-u_{m}^{t}\right|}^{\Lambda_{m}-1}\,H\,\left(S_{o\,m}|\Omega_{m}\right)}{\sum_{o=1}^{O}H\,\left(S_{o\,m}|\Omega_{m}\right)}$$

In formula (12), when *x* ≥ 0, *sign*(*x*) is equal to 1, otherwise it is equal to 0. $S\,o\,m∼T\,\left(I_{x}^{\theta }|u_{m}^{t},\sigma_{m}^{t},\Lambda_{m}^{t}\right)$ represents the random variable in the probability distribution $T\,\left(I_{x}^{\theta }|u_{m}^{t},\sigma_{m}^{t},\Lambda_{m}^{t}\right)$, *o* is the number of random variables *S*_*om*_. Note that *O* is a large integer, and *O* = 10^6^ is taken in this paper.


(13) $$\sigma_{m}^{t+1}={\left[\frac{\Lambda_{m}^{t}\,\beta \,\left(\Lambda_{m}^{t}\right)\,\sum_{x=1}^{X}\eta \,\left(z_{x\,m}\right)\,{\left|I_{x}^{\theta }-u_{m}^{t}\right|}^{\Lambda_{m}^{t}}}{\sum_{x=1}^{X}\eta \,\left(z_{x\,m}\right)\,\left(1+G\,m\right)}\right]}^{\frac{1}{\Lambda_{m}^{t}}}$$

Where *Gm* represents:


$$G_{m}$$

$$=\frac{\sum_{o=1}^{O}\left[-1+\Lambda_{m}^{t}\,\beta \,\left(\Lambda_{m}^{t}\right)\,{\left|S_{o\,m}-u_{m}^{t}\right|}^{\Lambda_{m}^{t}}\,{\left(\sigma_{m}^{t}\right)}^{-\Lambda_{m}^{t}}\right]\,H\,\left(S_{o\,m}|\Omega_{m}\right)}{\sum_{o=1}^{O}H\,\left(S_{o\,m}|\Omega_{m}\right)}$$

Under the condition that other parameters are fixed, use the Newton-Raphson method to estimate Λ_*m*_. Each iteration needs to solve the first and second derivatives of *Q*(ρ,ρ*^t^*) with respect to parameter Λ_*m*_. The next iteration value of Λ_*m*_ can be expressed as:


(15) $$\Lambda_{m}^{t+1}=\Lambda_{m}^{t}-{\frac{\partial Q\,\left(\rho ,\rho^{t}\right)}{\partial \Lambda_{m}}\,{\left[\frac{\partial Q^{2}\,\left(\rho ,\rho^{t}\right)}{\partial \Lambda_{m}^{2}}+\vartheta \right]}^{-1}|}_{\Lambda_{m}=\Lambda_{m}^{t}}$$

Where ϑ is the scale factor, and the derivative of *Q*(ρ,ρ*^t^*) with respect to Λ_*m*_ is given by:


$$\frac{\partial Q\,\left(\rho ,\rho^{t}\right)}{\partial \Lambda \,m}=-\sum_{x=1}^{X}\eta \,\left(z_{x\,m}\right)$$

$$\left[f\,\left(I_{x}^{\theta }|u_{m},\sigma_{m},\Lambda_{m}\right)-\frac{\int_{\partial }T\,\left(I_{x}^{\theta }|u_{m},\sigma_{m},\Lambda_{m}\right)\,f\,\left(I_{x}^{\theta }|u_{m},\sigma_{m},\Lambda_{m}\right)\,d\,x}{\int_{\partial }T\,\left(I_{x}^{\theta }|u_{m},\sigma_{m},\Lambda_{m}\right)\,d\,x}\right]$$

Where:


$$f\,\left(I_{x}^{\theta }|u_{m},\sigma_{m},\Lambda_{m}\right)=\left[\frac{1}{\Lambda_{m}}+\frac{3\,B\,G\,\left(\frac{1}{\Lambda_{m}}\right)-3\,B\,G\,\left(\frac{3}{\Lambda_{m}}\right)}{2\,\Lambda^{2_{m}}}\right]$$

$$-BG\left(\Lambda_{m}\right)|\frac{I_{x}^{\theta }-u_{m}}{\sigma_{m}}{|}^{\Lambda_{m}}\log|\frac{I^{\theta_{x}-u_{m}}}{\sigma_{m}}|-BG\left(\Lambda_{m}\right)\times$$

(17) $$\left[\frac{1}{2}\,\log\frac{\Gamma \,\left(\frac{3}{\Lambda_{m}}\right)}{\Gamma \,\left(\frac{1}{\Lambda_{m}}\right)}+\frac{B\,G\,\left(\frac{1}{\Lambda_{m}}\right)-3\,B\,G\,\left(\frac{3}{\Lambda_{m}}\right)}{2\,\Lambda_{m}}\right]\,{\left|\frac{I_{x}^{\theta }-u_{m}}{\sigma_{m}}\right|}^{\Lambda_{m}}$$

$$\int_{\partial }T\,\left(I_{x}^{\theta }|u_{m},\sigma_{m},\Lambda_{m}\right)\,f\,\left(I_{x}^{\theta }|u_{m},\sigma_{m},\Lambda_{m}\right)\,d\,x$$

(18) $$\approx \frac{1}{O}\,\sum_{o=1}^{O}f\,\left(S_{o\,m}|u_{m}^{t},\sigma_{m}^{t},\Lambda_{m}^{t}\right)\,H\,\left(S_{o\,m}|\Omega_{m}\right)$$

The second derivative of *Q*(ρ,ρ*^t^*) with respect to Λ_*m*_ is:


(19) $$\frac{\partial Q^{2}\,\left(\rho ,\rho^{t}\right)}{\partial \Lambda_{m}^{2}}=-\sum_{x=1}^{X}\eta \,\left(\phi_{x\,m}\right)$$

$$\left[\begin{array}{c} g\,\left(I_{x}^{\theta }|u_{m},\sigma_{m},\Lambda_{m}\right)+\frac{{\left(\int_{\partial }T\,\left(I_{x}^{\theta }|u_{m},\sigma_{m},\Lambda_{m}\right)\,f\,\left(I_{x}^{\theta }|u_{m},\sigma_{m},\Lambda_{m}\right)\,d\,x\right)}^{2}}{{\left(\int_{\partial }T\,\left(I_{x}^{\theta }|u_{m},\sigma_{m},\Lambda_{m}\right)\,d\,x\right)}^{2}}-\\ \frac{\int_{\partial }T\,\left(I_{x}^{\theta }|u_{m},\sigma_{m},\Lambda_{m}\right)\,\left[f^{2}\,\left(I_{x}^{\theta }|u_{m},\sigma_{m},\Lambda_{m}\right)+g\,\left(I_{x}^{\theta }|u_{m},\sigma_{m},\Lambda_{m}\right)\right]\,d\,x}{\int_{\partial }T\,\left(I_{x}^{\theta }|u_{m},\sigma_{m},\Lambda_{m}\right)\,d\,x} \end{array}\right]$$

Where,


$$\begin{array}{cc} \begin{array}{c} g\,\left(I_{x}^{\theta }|u_{m},\sigma_{m},\Lambda_{m}\right)\\ =\frac{\partial f\,\left(I_{x}^{\theta }|u_{m},\sigma_{m},\Lambda_{m}\right)}{\partial \Lambda_{m}}\\ =\left[\frac{-1}{\Lambda_{m}^{2}}-\frac{3\,B\,G\,\left(\frac{1}{\Lambda_{m}}\right)}{2\,\Lambda_{m}^{4}}-\frac{3\,B\,G\,\left(\frac{1}{\Lambda_{m}}\right)}{\Lambda_{m}^{3}}+\frac{9\,B\,G\,\left(\frac{3}{\Lambda_{m}}\right)}{2\,\Lambda_{m}^{4}}+\frac{3\,B\,G\,\left(\frac{3}{\Lambda_{m}}\right)}{\Lambda_{m}^{3}}\right]\\ -\beta \,\left(\Lambda_{m}\right)\,{\left|\frac{I_{x}^{\theta }-u_{m}}{\sigma_{m}}\right|}^{\Lambda_{m}}\,{\left(\log\left|\frac{I_{x}^{\theta }-u_{m}}{\sigma_{m}}\right|\right)}^{2}-\;\left(20\right)\\ \beta \,\left(\Lambda_{m}\right)\times {\left[\begin{array}{c} \frac{1}{2}\,l\,o\,g\,\frac{\Gamma \,\left(\frac{3}{\Lambda_{m}}\right)}{\Gamma \,\left(\frac{1}{\Lambda_{m}}\right)}+\frac{B\,G\,\left(\frac{1}{\Lambda_{m}}\right)-3\,B\,G\,\left(\frac{3}{\Lambda_{m}}\right)}{2\,\Lambda_{m}}+\\ \frac{-B\,G^{\prime }\,\left(\frac{1}{\Lambda_{m}}\right)+9\,B\,G^{\prime }\,\left(\frac{3}{\Lambda_{m}}\right)}{2\,\Lambda_{m}^{3}} \end{array}\right]}^{2}\\ {\left|\frac{I_{x}^{\theta }-u_{m}}{\sigma_{m}}\right|}^{\Lambda_{m}}-\beta \,\left(\Lambda_{m}\right)\times \left[\frac{1}{2}\,\log\frac{\Gamma \,\left(\frac{3}{\Lambda_{m}}\right)}{\Gamma \,\left(\frac{1}{\Lambda_{m}}\right)}+\frac{B\,G\,\left(\frac{1}{\Lambda_{m}}\right)-3\,B\,G\,\left(\frac{3}{\Lambda_{m}}\right)}{2\,\Lambda_{m}}\right]\\ {\left|\frac{I_{x}^{\theta }-u_{m}}{\sigma_{m}}\right|}^{\Lambda_{m}}\,\log\left|\frac{I_{x}^{\theta }-u_{m}}{\sigma_{m}}\right| \end{array} & \end{array}$$

$$\int_{\partial }T\,\left(I_{x}^{\theta }|u_{m},\sigma_{m},\Lambda_{m}\right)$$

$$\left[f^{2}\,\left(I_{x}^{\theta }|u_{m},\sigma_{m},\Lambda_{m}\right)+g\,\left(I_{x}^{\theta }|u_{m},\sigma_{m},\Lambda_{m}\right)\right]\,d\,x$$

$$\;\approx \frac{1}{O}\,\left[\sum_{o=1}^{O}f^{2}\,\left(S_{o\,m}|u_{m}^{t},\sigma_{m}^{t},\Lambda_{m}^{t}\right)+f\,\left(s_{o\,m}|u_{m}^{t},\sigma_{m}^{t},\Lambda_{m}^{t}\right)\right]$$

$$\,H\,\left(s_{o\,m}|\Omega_{m}\right)\;\left(21\right)$$

Finally, update the estimate of the prior probability $\tau_{m}^{t+1}$ that can be expressed as:


(22) $$\tau_{m}^{t+1}=\frac{1}{X}\,\sum_{x=1}^{X}\eta \,\left(z_{x\,m}\right)$$

### Motion Parameters Estimation

Optimize the corresponding parameter θ by deriving the result of *Q*(ρ,ρ*^t^*) to θ as 0:


(23) $$\frac{\partial Q\,\left(\rho ,\rho^{t}\right)}{\partial \theta }=0$$

In order to find the appropriate model movement parameter θ to satisfy the equation (23), introduce a small movement increment $\tilde{\theta }$ and replace θ with as the estimated parameter. The following is obtained by using approximate linear space transformation:


(24) $$I_{x}^{\theta +\tilde{\theta }}=I_{x}^{\theta }+\frac{\partial I_{x}^{\theta^{T}}}{\partial \theta }\,\tilde{\theta }$$

Incorporate formula (23) into formula (24) and the following can be obtained:


$$\left\{\sum_{x=1}^{X}\left[\sum_{m=1}^{M}\eta \,\left(z_{x\,m}\right)\,\Lambda_{m}\,\beta \,\left(\Lambda_{m}\right)\,\frac{\partial I_{x}^{\theta }}{\partial \theta }\,{\left(\sigma_{m}^{t+1}\right)}^{-1}\,\frac{\partial I_{x}^{\theta }{}^{T}}{\partial \theta }\right]\right\}\,\tilde{\theta }$$

$$=-\sum_{x=1}^{X}\left[\sum_{m=1}^{M}\eta \,\left(z_{x\,m}\right)\,\Lambda_{m}\,\beta \,\left(\Lambda_{m}\right)\,\frac{\partial I_{x}^{\theta }}{\partial \theta }\,{\left(\sigma_{m}^{t+1}\right)}^{-1}\,\left(I_{x}^{\theta }-u_{m}^{t+1}\right)\right]$$

The optimization of the registration parameters can be achieved by solving the movement increment $\tilde{\theta }$ in equation (25).

### Implementation

**FIGURE 1:**
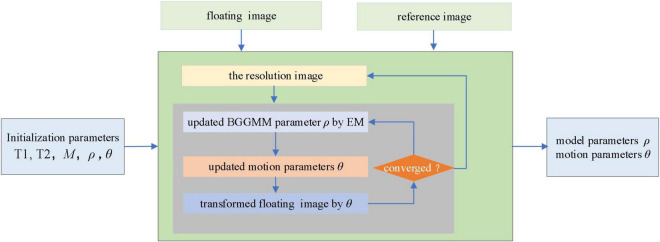
Flowchart of medical image registration.

In summary, the proposed image registration algorithm based on the BGGMM is shown in Algorithm 1 and Figure 1. This paper regards *M* BGG distribution components in the joint intensity scatter plot of the registered image as *M* clusters, uses the k-mean method to find the cluster centers and compares parameter initialization of the BGGMM model. This paper initializes Λ_*m*_ = 2. Secondly, this paper also utilizes multi-resolution image registration, and the resolutions are set [0.1 0.2 1], respectively. The image is first registered at low resolution and then high resolution, and the registration result at each resolution can be used as the next resolution registration. Therefore, the calculation time can be reduced, and the algorithm convergence can be accelerated in the iterative process of the proposed algorithm.

**Algorithm 1:** Description of algorithm for medical image registration based on BGGMM.

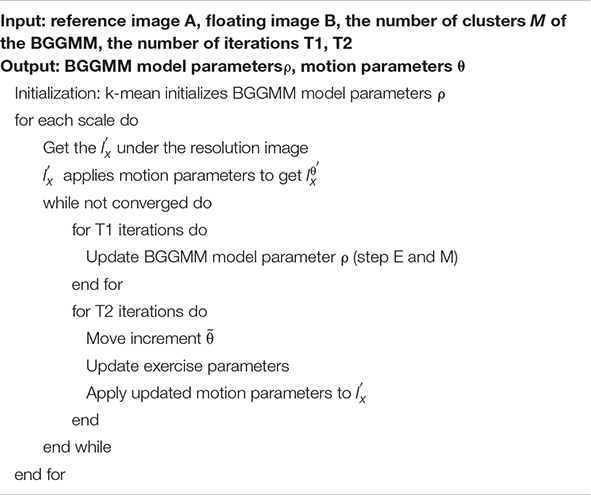

The EM algorithm is first used to estimate the BGGMM model parameter ρ on the joint intensity scatter plot. After the optimal BGGMM model parameter ρ is estimated for T1 times, the motion adjustment is performed. This paper introduces a small movement increment and iterates T2 times to update the motion parameters, ensuring the optimal parameters are obtained. Finally, iterate repeatedly until convergence to achieve image registration.

## Experiment

The computer environment of experiments in this paper is Intel(R) Core (TM) i5-7300HQ CPU @ 2.50 GHz with 8 GB RAM, while the operating system is 64-bit Windows 10.0. All simulations are implemented using MATLAB R2020b.

The mutual information method (MI) (Lu et al., 2008), the enhanced correlation coefficient (ECC) (Evangelidis and Psarakis, 2008) and the ensemble registration approach (ER) (Orchard and Mann, 2009) are compared to evaluate the performance of the proposed method. The average pixel displacement (PAD) (Li et al., 2016) is used as a registration error to objectively measure the performance of different approaches. In the successful registration case, the value of the PAD is zero. The larger the PAD, the more significant deviation and the lower registration accuracy. If PAD is greater than 3, the registration is considered to have failed.

MURA (Rajpurkar et al., 2017) and Altas (Yu and Zheng, 2016) public image data sets are used to verify the performance of these methods. Details about two image datasets and experiments are reported, as shown in Table 1, where the bold values indicate the best results. The *t*-test is used to test the significance of the difference between the PAD results of the BGGMM method and the other three registration methods in image registration on public data sets. *P* < 0.05 means the difference is statistically significant, and the comparison results are summarized in Table 2. Both in the MURA and Atlas data sets, the PAD results of the BGGMM method were minor, and the differences were statistically significant compared to the PAD results of the ECC and ER methods (*P* < 0.05). In the MURA data set, the difference between the PAD results of the BGGMM method and the MI method was not statistically different (*P* > 0.05). However, in the Atlas dataset, the PAD results of the BGGMM method were smaller than those of the MI method, and the difference was statistically significant (*P* < 0.05).

**TABLE 1:** The pad results of image registration on public data sets.

Method/dataset	Public dataset	
	MURA images	Atlas images
MI	1.9162	0.7168
ECC	8.1494	10.7606
ER	6.5182	9.1342
Proposed method	**0.2271**	**0.6801**

*The bold values indicated the best results.*

**TABLE 2:** The *t*-test results of the pad results of BGGMM versus other image registration methods on public data sets.

Database	Method		*p*-value
MURA	BGGMM	MI	0.132
		ECC	0.000
		ER	0.000
Atlas	BGGMM	MI	0.034
		ECC	0.001
		ER	0.000

### Musculoskeletal Radiographs Dataset

**FIGURE 2:**
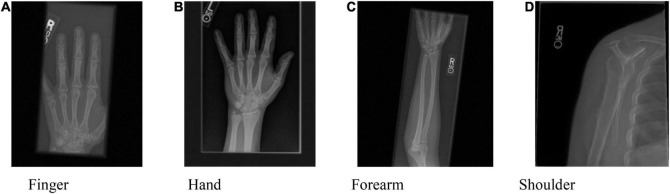
One slice of the MURA dataset. **(A)** Finger, **(B)** Hand, **(C)** Forearm, and **(D)** Shoulder.

The proposed approach is tested on an ensemble of MURA images. The test set is from the Large Dataset for Abnormality Detection in Musculoskeletal Radiographs (MURA) project’s training data set. One slice of this dataset is depicted in Figure 2. The initial image to be registered is generated by random translation and rotation transformation, and the pixel and angle transformation parameters ranges are [–20, 20] and [–10, 10], respectively. This paper sets *M* = 6, that is, the number of BGG distribution components in the initial model is 6. The MURA dataset included 12,173 patients, 14,863 studies, and 40,561 multi-view radiographs. Each study belonged to one of the seven standard upper limb radiology study types: fingers, elbows, forearms, hands, humerus, shoulders, and wrists. Each study was manually marked as normal or abnormal by the radiologist.

**FIGURE 3:**
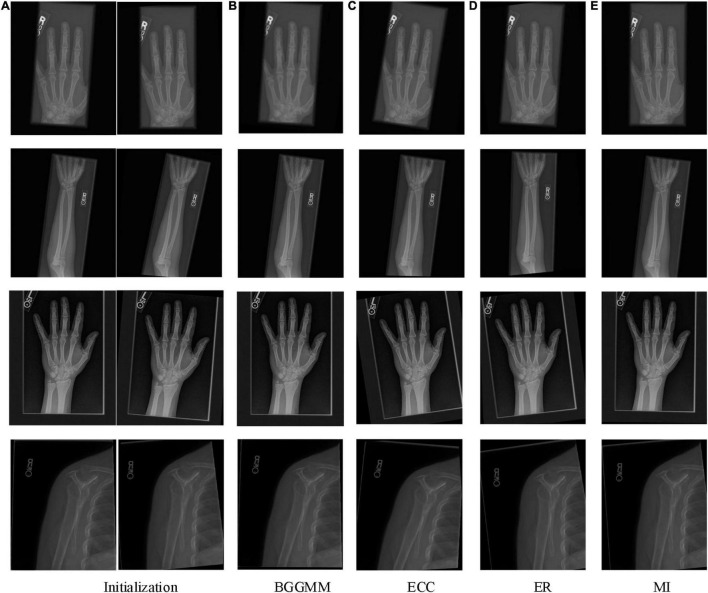
The registration results of four methods in four skeleton images of MURA dataset. **(A)** Initialization. **(B)** BGGMM. **(C)** ECC. **(D)** (ER). **(E)** MI.

The PAD values of the MURA dataset are summarized in the first column of Table 1. The average registration error of the proposed BGGMM method is significantly lower than other methods. The BGGMM method is more advantageous in edge retention and information content of source images. The registration results of the four methods are shown in Figure 3, which register the source image and transform the image with rotation and translation. In these four methods, registration is performed to the source image, and rotation, translation and transformation is performed to the image. Figure 3A shows the source image and the image to be registered.

**FIGURE 4:**
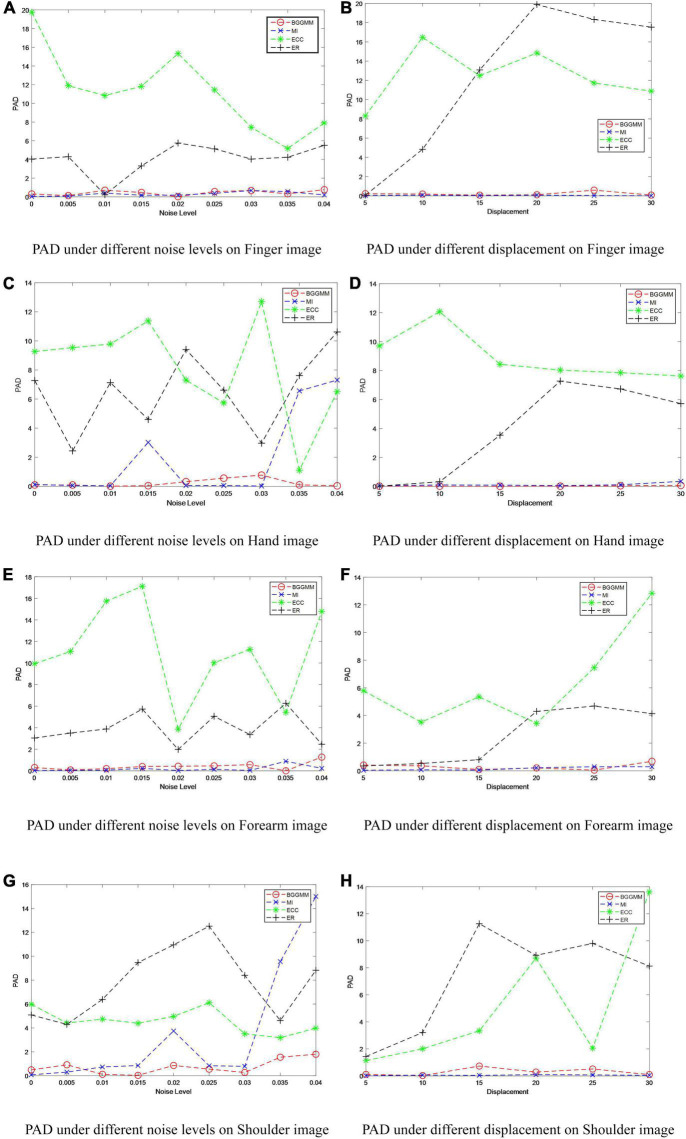
PAD of different methods under different noise levels and different displacements in MURA dataset. **(A)** PAD under different noise levels on Finger image. **(B)** PAD under different displacement on Finger image. **(C)** PAD under different noise levels on Hand image. **(D)** PAD under different displacement on Hand image. **(E)** PAD under different noise levels on Forearm image. **(F)** PAD under different displacement on Forearm image. **(G)** PAD under different noise levels on Shoulder image. **(H)** PAD under different displacement on Shoulder image.

With different noise levels, Gaussian noise is used as the independent variable in finger images of the experiment, and the noise level increases incrementally to test the performance of BGGMM. The mean value of Gaussian noise is 0, and the variance ranges from 0 to 0.04. As shown in Figure 4A, the excellent registration performance of several comparison algorithms can be observed. Among them, the registration error of the ER algorithm is the largest. The registration error of the BGGMM algorithm is lower than other methods under different noise levels.

The registration performance of the algorithm on Finger images is also tested under different displacement situations, as shown in Figure 4B. The displacement is added by moving the image *t* pixels horizontally and vertically, where the change range of *t* is 0–30, that is, the variation of the horizontal axis in Figure 4B. It is not difficult to see that the registration performance of this algorithm is better than other algorithms under different displacements. Among them, the ECC algorithm has poor anti-displacement interference, which is regarded as a registration failure. The ER algorithm has a good registration effect under the condition of small displacement. The BGGMM algorithm has the best performance when the change in displacement is large. Similarly, Figures 4C–H show the PAD value of different methods on Hand images, Forearm images, and Shoulder images under different noise levels and different displacements. The proposed method has the lowest registration error and the best registration performance.

### Altas Dataset

**FIGURE 5:**
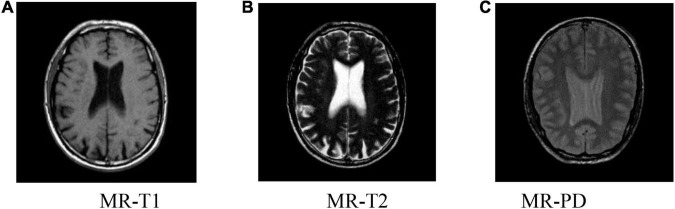
Brain slice images from the Atlas dataset. **(A)** MR-T1, **(B)** MR-T2, **(C)** MR-PD.

Altas dataset is a multimodal dataset that includes more than 13,000 MRI and CT images of patients with brain diseases. Among them, MRI images have images with T1, T2, and PD weights. At the same time, it also includes the lesion images of patients with different lesion times. The image in which the MRI has T1, T2, and PD weights is selected, as shown in Figure 5. The initial image to be registered is generated by random translation and rotation transformation, and the pixel and angle transformation parameters ranges are [–20, 20] and [–10, 10], respectively. This paper sets *M* = 6, that is, the number of BGG distribution components in the initial model is 6.

**FIGURE 6:**
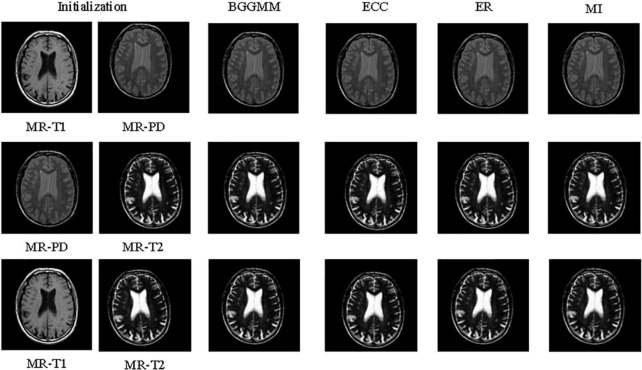
The registration results of four methods in the brain images of Altas dataset.

The PAD values of Altas dataset are summarized in the second column of Table 1. The average registration error of the proposed BGGMM method is significantly lower than other methods. The BGGMM method has an advantage in preserving the edge information of the source image. The registration results of the four methods are shown in Figure 6. In these four methods, two different modality images are used to register separately.

**FIGURE 7:**
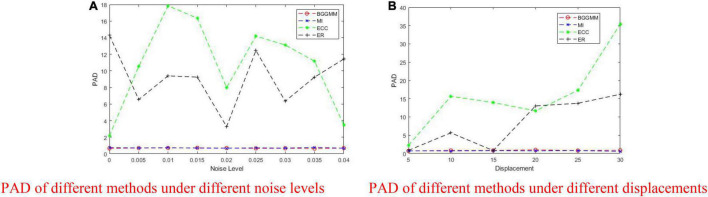
PAD of BGGMM, ECC, ER, and MI methods under different noise levels and different displacements. **(A)** PAD of different methods under different noise levels. **(B)** PAD of different methods under different displacements.

The registration performance of BGGMM, ECC, and ER methods is tested under different Gaussian noises. According to the registration results in Figure 7A, the comparison of registration effects under different Gaussian noises can be obtained. The mean value of Gaussian noise is 0, and the variance ranges from 0 to 0.04. Among them, the registration error of the ECC algorithm is the largest. The PAD value of other algorithms mentioned above in this experiment is greater than 3, which is regarded as registration failures. The BGGMM algorithm has the lowest PAD value and has good registration performance.

As shown in Figure 7B, the displacement is added by moving the image *t* pixels horizontally and vertically, where the change range of *t* is 0–30. When the displacement changes considerably, the error generated by the ER algorithm becomes larger and exceeds the effective range. As the change in displacement increases, the PAD value of our BGGMM algorithm is still unaffected, always maintaining a low level and performing better among the four algorithms.

## Conclusion

A medical registration method based on a BGGMM is proposed in this paper. Firstly, a BGGMM is applied to model the joint intensity vector distribution of the medical image. The proposed approach then formulates the model as an ML framework and estimates the parameters of models applying an EM algorithm. The experimental results indicate that the proposed BGGMM significantly improves registration performances on medical images compared with benchmark methods. The effect of this method is more pronounced when dealing with source images with more interference information and larger offsets. In the future, the research on medical image fusion will be carried out based on BGGMM image registration, which will provide convenience for medical image analysis.

## Data Availability Statement

The original contributions presented in this study are included in the article/supplementary material, further inquiries can be directed to the corresponding author/s.

## Author Contributions

YX and HZ conceived and designed the study. JW and KX conducted most of the experiments and data analysis and wrote the manuscript. KC, RL, and RN participated in collecting materials and assisting in drafting manuscripts. All authors reviewed and approved the manuscript.

## Conflict of Interest

The authors declare that the research was conducted in the absence of any commercial or financial relationships that could be construed as a potential conflict of interest.

## Publisher’s Note

All claims expressed in this article are solely those of the authors and do not necessarily represent those of their affiliated organizations, or those of the publisher, the editors and the reviewers. Any product that may be evaluated in this article, or claim that may be made by its manufacturer, is not guaranteed or endorsed by the publisher.
